# Germline genetic testing among patients with pancreatic adenocarcinoma: A Pancreatic Cancer Action Network patient survey

**DOI:** 10.1002/cncr.70446

**Published:** 2026-05-12

**Authors:** Udhayvir S. Grewal, Rishi R. Patel, Bradley T. Loeffler, Sydney Rathjens, Kawther Abdilleh, Fatima Zelada‐Arenas, Nicholas J. Hornstein, Timothy J. Brown, Seth J. Concors, Naomi H. Fei, Saima Sharif, Chandrikha Chandrasekharan

**Affiliations:** ^1^ Winship Cancer Institute of Emory University Atlanta Georgia USA; ^2^ Holden Comprehensive Cancer Center Iowa City Iowa USA; ^3^ University Hospitals Cleveland Medical Center Cleveland Ohio USA; ^4^ Pancreatic Cancer Action Network El Segundo California USA; ^5^ Northwell Health Cancer Institute New York New York USA; ^6^ Simmons Comprehensive Cancer Center UT Southwestern Medical Center Dallas Texas USA; ^7^ The University of Texas MD Anderson Cancer Center Houston Texas USA

**Keywords:** genetic counseling, germline genetic testing, inequities, pancreatic cancer, precision oncology

## Abstract

**Background:**

Approximately 10%–15% of patients with pancreatic ductal adenocarcinoma (PDAC) harbor pathogenic germline genetic alterations with direct therapeutic and hereditary cancer implications, leading to guideline recommendations for universal germline genetic testing regardless of family history.

**Methods:**

The authors conducted a cross‐sectional electronic survey in collaboration with the Pancreatic Cancer Action Network (PanCAN). Surveys were distributed October–December 2024 to registry participants. Primary outcomes included being offered and completion of germline genetic testing. Secondary outcomes included genetic counseling, mutation results, and cascade testing among first‐degree relatives (FDR). Multivariable logistic regression was used to identify factors associated with testing offer and completion.

**Results:**

Among 1046 respondents, 66.2% were offered germline genetic testing and 69.2% completed testing. Black race, lack of insurance, unknown stage at diagnosis, and treatment at community practices were associated with lower odds of being offered testing. Completion of testing was lower among Black and Hispanic participants, uninsured individuals, and those treated in community settings, whereas patients with stage IV disease had the highest odds of completion. Among tested participants, 23.2% had a pathogenic germline variant, most commonly *BRCA2*, *ATM*, and *BRCA1*. Only 61.7% of mutation‐positive respondents reported cascade testing in an FDR. Receipt of genetic counseling was associated with higher rates of cascade testing (*p* < .01).

**Conclusions:**

Universal germline genetic testing in PDAC remains incompletely implemented, with persistent inequities. Lack of discussion or offer of testing represents a key missed opportunity, underscoring the need for targeted interventions and expanded access.

## Abstract 2

Universal germline genetic testing in pancreatic adenocarcinoma remains underused, with significant disparities by race, insurance status, and care setting affecting both offer and completion rates. Despite a high prevalence of actionable mutations, missed opportunities in testing and counseling limit cascade testing, highlighting the need for targeted interventions to improve equitable implementation.

## INTRODUCTION

Pancreatic ductal adenocarcinoma (PDAC) remains one of the leading causes of cancer‐related mortality in the United States and worldwide. Most patients present with advanced or metastatic disease, precluding curative resection. Recent advances in systemic therapy have demonstrated modest improvements in survival outcomes in randomized clinical trials, however the overall prognosis remains poor.^1^ Recent clinical trials have also demonstrated a role for new and expanded therapeutic options among patients with PDAC harboring germline genetic alterations such as *BRCA1/2* and *PALB2*.^2^ ^,^ ^3^ Thus, there is a growing emphasis on germline genetic testing to aid in personalizing therapeutic options for patients with cancer.^4^ Approximately 10% of patients with PDAC may harbor pathogenic germline variants, most frequently in genes involved in homologous recombination repair (HRR), including *BRCA1*, *BRCA2*, *PALB2*, and *ATM*. Other alterations include Lynch syndrome (*MLH1*, *MSH2*, *MSH6*, *PMS2*, and *EPCAM*), Puetz Jeghers syndrome (*STK11*), hereditary pancreatitis (*PRSS1*), and germline *CKDN2A* mutations.^5^ The relatively high prevalence of these germline genetic alterations including in patients without documented prior family history of cancer led to the recommendation for universal germline genetic testing among patients with PDAC. Current National Comprehensive Cancer Network (NCCN) and American Society of Clinical Oncology (ASCO) guidelines recommend germline testing for all patients with PDAC, regardless of family history.^6^ ^,^ ^7^

The identification of pathogenic germline genetic alterations carries direct therapeutic implications. Patients with HRR‐deficient tumors demonstrate increased sensitivity to platinum‐based chemotherapy and may benefit from PARP inhibition as maintenance therapy. The phase 3 Pancreas Cancer Olaparib Ongoing trial established the role of PARP inhibitors in PDAC by demonstrating that maintenance therapy with olaparib significantly prolonged progression‐free survival (compares to placebo), among patients with germline *BRCA1/2* mutations whose disease had not progressed after first‐line platinum‐based chemotherapy.^2^ Similarly, a wealth of clinical data support superior outcomes with platinum‐based regimens in patients with PDAC harboring germline HRR alterations, reinforcing the predictive value of germline profiling for therapeutic selection.^3^ ^,^ ^8^ In addition to guiding treatment decisions, germline testing has significant implications for hereditary cancer screening and cancer prevention. Cascade testing of first‐degree relatives (FDR) following the identification of a germline pathogenic variant may enable timely detection, surveillance, or appropriate risk reduction strategies.^9^

Existing data show that real‐world uptake of germline testing among all patients with cancer including PDAC remains low or suboptimal.^10^ ^–^ ^12^ Barriers to guideline recommended universal germline testing remain poorly understood. Data from other cancers such as breast cancer, where germline genetic testing is also widely recommended, indicates that lack of provider and/or patient awareness, limited access to genetic counseling, and financial concerns may act as key barriers.^13^ Given these data, we sought to evaluate patient‐reported access to and experiences with germline genetic testing among individuals with PDAC, and to identify factors associated with inequities in testing offer, uptake, and genetic counseling.

## MATERIALS AND METHODS

This was a prospective electronic survey study conducted in collaboration with Pancreatic Cancer Action Network (PanCAN), a United States‐based nonprofit organization that provides patient/caregiver support, conducts community outreach, and advocates for pancreatic cancer research funding. PanCAN maintains an online pancreatic cancer‐specific registry where patients and caregivers may self‐report data related to their diagnosis and treatment. PanCAN maintains an institutional review board‐approved, secure registry, launched in 2015 and comprising over 2000 participants as of 2024. Additionally, PanCAN has a list of patients with PDAC and their caregivers (self‐identified) who have signed up for receiving communications from the organization. It captures self‐reported sociodemographic, clinical, treatment, and outcomes data from patients and caregivers. Participation in the registry is voluntary and involves informed consent that is obtained before enrollment.

**FIGURE 1:**
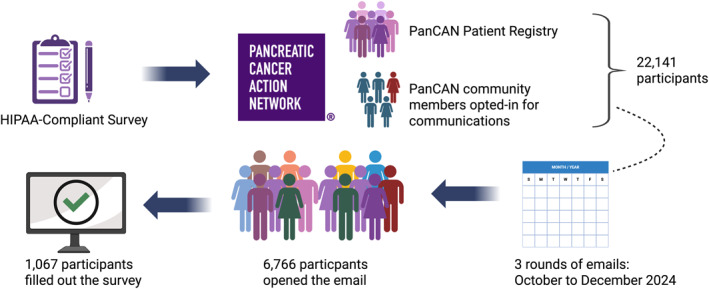
Survey workflow and methodology. Flow diagram illustrating the dissemination and completion of a Health Insurance Portability and Accountability Act of 1996‐compliant survey administered in collaboration with the Pancreatic Cancer Action Network (PanCAN). Survey invitations were sent to 22,141 individuals within the PanCAN patient registry and community members who had previously opted in to receive communications. Across three email rounds distributed between October and December 2024, 6766 recipients opened the email, and 1067 participants completed the survey.

For the purpose of this study, a Health Insurance Portability and Accountability Act of 1996‐compliant electronic survey was sent to registered patients and caregivers that had opted in to participate in surveys. The survey was distributed in three rounds between October and December 2024 to patients who were a part of the registry as well as patients and caregivers in the PanCAN email list (Figure 1). Enrollment was voluntary and independent profiles were created through PanCAN’s website. This study was deemed exempt from review by the University of Iowa institutional review board, as it involved analysis of de‐identified, voluntarily submitted data. Survey responses were captured on REDCAP database.

We developed a cross‐sectional survey assessing self‐reported experiences with genetic testing and counseling. Questions covered self‐reported demographics (age at diagnosis, gender, and residence—rural vs. urban), clinical factors (e.g., stage at diagnosis and type of treatment facility), genetic testing details (awareness, offer, uptake, counseling, and financial burden), as well as personal or family history of cancer and cascade testing. Respondents were included if they self‐identified as pancreatic cancer patients and answered questions about genetic testing offer and uptake. Those reporting treatment for pancreatic neuroendocrine tumors (e.g., lanreotide, peptide receptor radionuclide therapy, or Y90 liver embolization) were excluded.

Statistical analyses were performed using SAS version 9.4. The two primary outcomes were whether respondents were offered and whether they completed genetic testing. Secondary outcomes included genetic counseling, financial burden, mutation results, and cascade testing among FDR. Univariate logistic regression assessed associations between predictors and outcomes. For each outcome, a multivariate model was built to include all characteristics that were significant on univariate analysis. In a subset of participants who tested positive for inherited alterations, the association between genetic counseling and family cascade testing was analyzed using a χ^2^ test. All analyses were two‐sided with significance set at α = 0.05.

## RESULTS

The survey email was circulated among 22,141 participants, and 6766 participants (30.6%) opened the email. Of these, 1067 participants (15.8%) responded and 1046 were included in the current analysis (Figure 1). Baseline characteristics of the entire cohort are presented in Table 1. Of 1046 patients, 692 (66.2%) participants were offered, 89 (8.5%) did not remember being offered, and 265 (25.3%) were not offered germline genetic testing after diagnosis. Ultimately, a total of 724 participants (69.2%) underwent germline genetic testing. In patients who were not offered testing but did complete testing (*N* = 63), 18 (28.6%) had a personal history of prior non‐PDAC cancer diagnosis.

**TABLE 1:** Baseline characteristics of patients included in the survey (*N* = 1046).

Variable	Level	No. (%), *N* = 1046
Gender	Male	446 (42.6)
	Female	597 (57.1)
	Transgender	1 (0.1)
	Prefer not to answer	2 (0.2)
Race	Asian or Pacific Islander	28 (2.7)
	Black or African American	52 (5.0)
	Hispanic or Latino	47 (4.5)
	Native American or Alaskan Native	5 (0.5)
	White or Caucasian	894 (85.6)
	Multiracial	6 (0.6)
	A race/ethnicity not listed here	13 (1.2)
	Missing	1
Area of residence	Urban	658 (63.0)
	Rural	265 (25.4)
	Other	122 (11.7)
	Missing	1
Insurance type	Medicaid	29 (2.8)
	Medicare	406 (38.9)
	Veterans’ Health Administration	13 (1.2)
	Private	501 (47.9)
	Other	84 (8.0)
	None	12 (1.1)
	Missing	1
Stage at diagnosis	Stage I	256 (24.5)
	Stage II	258 (24.7)
	Stage III	184 (17.6)
	Stage IV	246 (23.5)
	I do not know	102 (9.8)
Received surgery for pancreatic cancer	No	316 (30.2)
	Yes	730 (69.8)
Received chemotherapy for pancreatic cancer	No	146 (14.0)
	Yes	900 (86.0)
Received radiation therapy for pancreatic cancer	No	674 (64.4)
	Yes	372 (35.6)
Received immunotherapy for pancreatic cancer	No	966 (92.4)
	Yes	80 (7.6)
Received other therapy for pancreatic cancer	No	985 (94.2)
	Yes	61 (5.8)
Treatment facility type	Academic/teaching hospital	570 (54.5)
	Small community practice	72 (6.9)
	Large community practice	403 (38.6)
	Missing	1
Family history of cancer (first degree)	No	335 (32.0)
	Yes	664 (63.5)
	I do not know	47 (4.5)

On multivariate analysis, race (*p* = .04), insurance type (*p* < .01), stage at diagnosis (*p* < .01), and treatment facility type (*p* < .01) were significantly associated with the odds of being offered germline genetic testing following PDAC diagnosis. Black patients had 47% decreased odds of being offered genetic testing compared with White patients (odds ratio [OR], 0.53; 95% CI, 0.29–0.96). Uninsured patients had 83% lower odds of being offered testing compared with Medicare beneficiaries (OR, 0.17; 95% CI, 0.04–0.66), whereas patients with “other” insurance types (OR, 2.08; 95% CI, 1.18–3.68) or private insurance (OR, 1.43; 95% CI, 1.07–1.92) had significantly increased odds of being offered testing. Patients who did not know their cancer stage had 66% decreased odds of being offered genetic testing relative to stage I patients (OR, 0.34; 95% CI, 0.21–0.55). Patients with stage II/III disease had higher odds of being offered testing compared with stage I (OR, 1.30; 95% CI, 0.93–1.82), although this did not reach statistical significance. In contrast, those with stage IV disease had 1.80‐times higher odds of being offered testing compared with stage I (95% CI, 1.21–2.69). Finally, patients treated in community practices had significantly lower odds of being offered testing than those treated at academic/teaching hospitals (OR, 0.63; 95% CI, 0.48–0.83) (Table 2).

**TABLE 2:** Multivariate analysis investigating predictors of offer of germline genetic testing among patients with PDAC.

Covariate	Level	*N* ^a^	Odds of genetic testing offered		
			OR	95% CI	*p*
Race	Asian or Pacific Islander	28	0.91	0.39–2.14	0.04
	Black or African American	52	0.53	0.29–0.96	
	Hispanic or Latino	47	0.55	0.30–1.02	
	Other race/multiracial	24	0.46	0.19–1.10	
	White or Caucasian	892	Ref		
Insurance type	Medicaid	29	0.82	0.37–1.82	<.01
	None	12	0.17	0.04–0.66	
	Other	83	2.08	1.18–3.68	
	Private	500	1.43	1.07–1.92	
	Veterans’ Health Administration	13	0.30	0.09–1.02	
	Medicare	406	Ref		
Stage at diagnosis	I do not know	102	0.34	0.21–0.55	<.01
	Stage II/III	440	1.30	0.93–1.82	
	Stage IV	246	1.80	1.21–2.69	
	Stage I	255	Ref		
Treatment facility type	Large/small community practice	473	0.63	0.48–0.83	<.01
	Academic/teaching hospital	570	Ref		

Abbreviations: OR, odds ratio; PDAC, pancreatic ductal adenocarcinoma.

^a^ Number of observations in the original data set = 1046. Number of observations used = 1043.

On multivariable analysis evaluating completion of testing, Black (OR, 0.42; 95% CI, 0.23–0.78) and Hispanic (OR, 0.48; 95% CI, 0.26–0.92) participants were significantly less likely to complete testing than White participants. Compared to Medicare beneficiaries, uninsured participants had 89% lower odds of completion (OR, 0.11; 95% CI, 0.03–0.44), and those receiving care through the Veterans Health Administration also had reduced odds (OR, 0.26; 95% CI, 0.08–0.92). Conversely, participants with private insurance were more likely to complete testing (OR, 1.52; 95% CI, 1.12–2.07). Participants with stage IV disease had the highest odds of completing testing (OR, 2.21; 95% CI, 1.45–3.36), followed by those with stage II/III disease (OR, 1.56; 95% CI, 1.10–2.21), whereas participants who did not know their stage were substantially less likely (OR, 0.30; 95% CI, 0.18–0.49). Those treated in community practices were also significantly less likely to complete testing than participants treated at academic centers (OR, 0.60; 95% CI, 0.45–0.80) (Table 3).

**TABLE 3:** Multivariate analysis investigating predictors of undergoing germline genetic testing among patients with PDAC.

Covariate	Level	*N* ^a^	Odds of genetic testing completed		
			OR	95% CI	*p*
Race	Asian or Pacific Islander	28	1.42	0.52–3.86	0.01
	Black or African American	52	0.42	0.23–0.78	
	Hispanic or Latino	47	0.48	0.26–0.92	
	Other race/multiracial	24	0.68	0.27–1.71	
	White or Caucasian	892	Ref		
Insurance type	Medicaid	29	0.71	0.32–1.61	<.01
	None	12	0.11	0.03–0.44	
	Other	83	1.69	0.95–3.00	
	Private	500	1.52	1.12–2.07	
	Veterans’ Health Administration	13	0.26	0.08–0.92	
	Medicare	406	Ref		
Stage at diagnosis	I do not know	102	0.30	0.18–0.49	<.01
	Stage II/III	440	1.56	1.10–2.21	
	Stage IV	246	2.21	1.45–3.36	
	Stage I	255	Ref		
Treatment facility type	Large/small community practice	473	0.60	0.45–0.80	<.01
	Academic/teaching hospital	570	Ref		
Ever diagnosed with breast cancer	Yes	75	0.66	0.39–1.12	0.12
	No	968	Ref		
Family history of ovarian cancer	Yes	51	0.55	0.30–1.02	0.06
	No/do not know	992	Ref		

Abbreviations: OR, odds ratio; PDAC, pancreatic ductal adenocarcinoma.

^a^ Number of observations in the original data set = 1046. Number of observations used = 1043.

Among respondents who did not undergo testing (*n* = 322), the most commonly cited reason was that genetic testing or counseling was never discussed or offered (66.8%, *n* = 215). Only a small fraction reported insurance concerns (7.8%, *n* = 25), personal preference (3.1%, *n* = 10) or fear of the outcome (1.2%, *n* = 4). Approximately 8.4% (*n* = 27) patients already had a prior diagnosis of a germline genetic mutation and therefore did not undergo repeat genetic testing, whereas 16.1% (*n* = 52) patients quoted other reasons (Table 4). Notably, among patients who were not offered testing but did complete testing (*N* = 63), 18 (28.6%) had a personal history of cancer.

**TABLE 4:** Reasons reported for not undergoing germline genetic testing by survey respondents.

Reason for not getting tested	Response	No. (%), *N* = 322
Genetic testing or counselling was never discussed or offered to me	No	107 (33.2)
	Yes	215 (66.8)
I did not feel like getting tested	No	312 (96.9)
	Yes	10 (3.1)
I did not feel like it was important to get tested	No	311 (96.6)
	Yes	11 (3.4)
I was scared of getting tested/the outcomes of the testing	No	318 (98.8)
	Yes	4 (1.2)
The testing center was too far away from where I live	No	318 (98.8)
	Yes	4 (1.2)
I was afraid that my insurance would not cover the cost of genetic testing	No	297 (92.2)
	Yes	25 (7.8)
I have been tested already and have an inherited genetic mutation	No	295 (91.6)
	Yes	27 (8.4)
Other reason	No	270 (83.9)
	Yes	52 (16.1)

Among those (*n* = 724) who completed testing, 62.1% (*n* = 448) received formal genetic counseling, and 23.2% (*n* = 167) tested positive for an inherited mutation. The most common pathogenic variants identified were *BRCA2* (32.7%), *ATM* (15.2%), and *BRCA1* (9.7%). Notably, only 61.7% (*n* = 103) of those testing positive reported that a family member subsequently underwent genetic testing. Those that underwent genetic counseling were more likely to have cascade testing of a FDR completed versus those who did not undergo genetic counseling (67.7% vs. 38.2%, *p* < .01).

## DISCUSSION

In this national survey of patients with PDAC, we noted that fewer than 70% participants completed germline genetic testing despite guideline recommendations for universal testing. Testing access and uptake appeared to be significantly impacted by race, insurance status, stage at diagnosis, and treatment setting. Black patients, those lacking insurance coverage, and those receiving treatment at a nonacademic facility were less likely to complete testing, highlighting key barriers to implementation of guideline‐directed germline genetic testing. Notably, we also found that genetic counselling significantly increased the odds of cascade testing among FDR.

Our analysis is one of the first patient‐reported surveys in PDAC that adds to a growing body of evidence that indicates suboptimal implementation of universal germline genetic testing across cancer types.^14^ ^,^ ^15^ The exact rates of germline testing in PDAC range widely in literature from 20% to as high as 77% depending on the institution, patient population, time of the conduct of the study or the type of testing practice such as provider initiated referral versus mainstream universal testing.^16^ ^,^ ^17^ Our study did not have participants from a single institution or represent a specific pattern of testing and thus may be more representative of the general practice in a broader population. In our cohort of patients likely representing patients diagnosed after the 2018 NCCN guidelines, nearly 25% were still not offered germline genetic testing. Additionally, among those who did not undergo testing, 67% participants quoted not being offered as the primary reason rather than insurance or financial barriers. This continues to highlight provider and system‐based barriers such as health practitioner awareness, lack of time, lack of structured practices to implement guidelines, lack of access to testing, and counseling that lead to undertesting.^18^

Compared to White patients, Black patients were significantly less likely to be offered genetic testing and both Black and Hispanic patients were significantly less likely to complete genetic testing. This is consistent with existing literature that shows undertesting in racial and ethnic minorities. A large‐scale analysis of male veterans with prostate, pancreatic and breast cancer demonstrated that Black race was associated with significantly lower rates of germline genetic testing.^19^ Similar to the current analysis, a prior retrospective analysis also showed that Hispanic patients with pancreatic cancer were less likely to undergo germline genetic testing compared to non‐Hispanic patients, highlighting key inequities.^20^ A systematic review and meta‐analysis evaluating germline BRCA mutation testing specifically in PDAC also noted scarce testing in non‐White population^21^ These data highlight persistent race‐based inequities in health care access directly impacting the care of patients with cancer.^10^ When compared to Medicare beneficiaries, uninsured patients were at reduced odds of being offered and undergoing germline genetic testing, which may be explained by inability to cover out‐of‐pocket costs.^22^ Additionally, patients with Veterans’ Health Administration insurance coverage were at reduced odds of completing testing despite no differences in odds of being offered germline genetic testing. Although the trend could be also be attributed to other factors such as delays in care, overall clinical decline after diagnosis, personal preferences, etc., a prior analysis of veterans with prostate cancer reported that some veterans may decline germline testing out of fear of losing service‐connected benefits.^23^ Taken together, these findings highlight a missed opportunity for personalized therapies and cascade testing among veterans. We also noted that patients who received treatment at community practices were less likely to be offered, and complete germline genetic testing compared to academic centers. Several challenges such as a lack of infrastructure and resources, lack of time, and lack of easy access to genetic counselors may be potential causes of relatively lower uptake of germline genetic testing in patients receiving care in the community settings. These findings are noteworthy as majority of patients with cancer receive care in the community settings and highlight the need for targeted interventions to amplify germline testing among these patients.^24^ It is also interesting to note that those with stage IV cancer were more likely to be offered genetic testing compared to earlier stage disease despite the guidelines for universal testing regardless of the stage of the cancer. One possible explanation for this observation may be higher rates of testing in advanced‐stage disease due to the availability and greater need for therapeutic options in this group of patients. However, this also underscores a missed opportunity as patients with earlier stage disease receiving curative intent therapy may have a longer time available to undergo testing and follow other screening guidelines if a pathogenic mutation was detected

As previously discussed, approximately 10%–15% patients with PDAC may harbor germline genetic alterations, which not only has therapeutic relevance to the patient but may also facilitate cascade testing among FDR in the appropriate setting.^25^ ^,^ ^9^ Nearly 40% of patients who tested positive for a germline genetic alteration in our survey did not report a family member undergoing cascade testing. This is in concordance with existing data that shows that the uptake of cascade genetic testing among FDR remains low, approximately 15%–30%.^26^ ^,^ ^27^ Importantly, in our study, genetic counseling was significantly associated with greater odds of cascade testing among FDR. This underscores the important role that genetic counselors may play in facilitating cascade testing and hereditary cancer prevention among FDR of patients with PDAC.

In summary, our data emphasize the existing gap and ongoing need for targeted interventions to ensure the broader and effective implementation of guideline‐directed universal germline testing in PDAC. Burgeoning evidence suggests that models other than traditional germline counseling and testing directed by oncology physicians such as mainstreaming germline genetic testing carries the most potential to increase testing rates among patients with PDAC. This also ensures judicious use of resources such as genetic counseling to be directed to patients with a positive germline testing to facilitate education regarding cancer screening and cascade testing as appropriate. In this model, germline testing may be ordered by an oncologist, oncology team member, or a genetic counseling assistant. Limited pretest counseling can be provided, using educational materials developed by genetic counselors (e.g., videos, documents, or supplemental resources). A genetic counselor may participate in disclosing all test results and providing posttest counseling, although in some workflows oncologists refer only patients with pathogenic germline variants for genetic counseling, cascade testing, and subsequent follow‐up as appropriate.^9^ ^,^ ^28^ ^,^ ^29^ This also has the advantage of saving patients facing a significant illness the burden of extra visits leading to missed opportunity for testing. Other innovative models such as remote genetic counseling and testing or use of telephone based counseling and remote saliva kit testing especially in unaffected general population with PDAC affected first degree family members has yielded higher testing completion rates.^30^ However, such models may still not be ready for large scale implementation due to the limitations in testing coverage by insurance. Furthermore, such models of care delivery may still not benefit patients with limited health literacy or limited access to technology as well as the traditionally underserved minorities.

The current analysis has several limitations. We acknowledge that the patients in our study reporting completion of germline genetic testing may not fully reflect real‐world practice patterns. Our cohort was drawn from individuals engaged with an advocacy organization such as PanCAN, which may serve as a surrogate for higher health literacy, stronger disease awareness, and greater engagement with cancer‐related resources. Because the survey was administered online and only in English, respondents also represent a population with reliable internet access, basic technological proficiency, and English language fluency, further limiting generalizability. The survey is limited by recall bias that may directly impact the accuracy of the data reported by the respondents. Additionally, the reported positivity rate for germline genetic alterations likely includes both clinically actionable pathogenic variants and lower‐penetrance or secondary findings, which may explain the higher rate (23.2%) compared to prior reports (10%–15%). The survey also did not capture detailed information regarding the type of provider ordering genetic testing (e.g., oncologist vs. genetic counselor) or whether testing occurred via point‐of‐care versus referral pathways, which could have offered helpful insights for care delivery. Despite these limitations, our findings highlight persistent and clinically meaningful barriers to germline testing and provide a foundation for designing targeted interventions to broaden equitable access to genetic evaluation for all patients with pancreatic cancer.

In conclusion, our study is one of the first direct patient reported survey highlighting the suboptimal implementation of universal germline genetic testing guidelines among patients with PDAC. We also highlight key inequities based on race, insurance status, and treatment setting. The observation that the lack of discussion or offer of genetic testing was the primary reason in the majority not undergoing germline testing is a missed opportunity and highlights the need for increasing awareness and resources to maximize implementation of universal testing. Additionally, we also show that genetic counselling may be key for improving rates of cascade germline genetic testing among FDR. Future efforts should also prioritize targeted interventions to amplify testing in community settings, address race‐based inequities in health care access, and leverage mainstreaming workflows to maximize the implementation of guideline‐directed universal germline testing.

## AUTHOR CONTRIBUTIONS

**Udhayvir S. Grewal**: Conceptualization; visualization; methodology; data curation; writing–original draft; writing–review and editing. **Rishi R. Patel**: Methodology; data curation; writing–original draft; writing–review and editing. **Bradley T. Loeffler**: Methodology; formal analysis; writing–original draft; writing–review and editing. **Sydney Rathjens**: Conceptualization; writing–original draft; writing–review and editing. **Kawther Abdilleh**: Conceptualization; writing–original draft; writing–review and editing. **Fatima Zelada‐Arenas**: Writing–original draft; writing–review and editing. **Nicholas J. Hornstein**: Writing–original draft; writing–review and editing. **Timothy J. Brown**: Writing–original draft; writing–review and editing. **Seth J. Concors**: Writing–original draft; writing–review and editing. **Naomi H. Fei**: Writing–original draft; writing–review and editing. **Saima Sharif**: Writing–original draft; writing–review and editing. **Chandrikha Chandrasekharan**: Conceptualization; methodology; writing–original draft; writing–review and editing; supervision.

## CONFLICT OF INTEREST STATEMENT

Timothy J. Brown reports consulting fees from Astellas Pharma, Daiichi‐Sankyo, and Incyte. Chandrikha Chandrasekharan reports consulting fees from Adcendo and Exelixis Inc. Seth J. Concors reports consulting fees from RYZ; and fees for other professional activities from Curio Science. Udhayvir S. Grewal reports consulting fees from Boehringer Ingelheim; and fees for other professional activities from Exelixis. The other authors report no conflicts of interest.

## Data Availability

The data that support the findings of this study are available on request from the corresponding author. The data are not publicly available due to privacy or ethical restrictions.
